# CD Oxyanions as a Tool for Synthesis of Highly Anionic Cyclodextrin Polymers †

**DOI:** 10.3390/polym12122845

**Published:** 2020-11-29

**Authors:** Tomasz Girek, Kinga Koziel, Beata Girek, Wojciech Ciesielski

**Affiliations:** Faculty of Mathematics and Natural Science, Jan Dlugosz University in Czestochowa, Armii Krajowej Ave., 13/15, 42 201 Czestochowa, Poland; kingakoziel123@gmail.com (K.K.); b.girek@ajd.czest.pl (B.G.); wc@ajd.czest.pl (W.C.)

**Keywords:** cyclodextrin, cyclodextrin polymers, anionic polymers, oxyanions, HPSEC-MALLS-RI investigation

## Abstract

Water soluble highly anionic β-cyclodextrin-based polymers were synthesized by reaction between cyclodextrin oxyanion and pyromellitic anhydride. The synthesis method utilizes activation hydroxyl groups in anhydrous glucopyranosyl units (AGU) in the DMF solution with the use of NaH. In these conditions, like in the case of the cyclodextrin reactions in the highly alkaline media, there is a nucleophilic substitution of difunctional compounds, which develops a polymer network with various cyclodextrin substitution. Different molar ratios of the reagents were investigated in terms of molecular size, chemical structure and water solubility of the polymers. The separation of the polymer due to particle size by ultrafiltration process and HPSEC-MALLS-RI and MALDI-TOF MS measurements for molecular mass analysis were employed. The IR, H NMR, SEM, DSC and TG measurements were taken for the structural characterization of the polymers. Additionally, the solubility test and metal ion complexation processes were also investigated in a wide range of pH. These polymers could be used in several areas such as: improving the aqueous solubility of poor water-soluble molecules, removing heavy metals from waste water, protecting degradable substances or synthesizing new drug delivery systems.

## 1. Introduction

Polymeric CDs are a well-known class of CD compounds which are intensively studied because of their valuable properties; for this reason, their applications are promising in various agrochemical, environmental and industrial areas [1].

Many kinds of polymeric CDs are known; polymeric CD can be obtained through several methods. However, two methods are commonly used. In one way, CD molecules are attached as a pendent group on the other polymeric chain. It can be prepared by radical polymerization of the monofunctional CD monomers with double or triple bonds in unsaturated CD moiety. It could be acroylyl cyclodextrin (CD-A) [2] or *N*-acroylyl-6-aminocaprocyclodextrin (CD-NAC) [3]. Atom transfer radical polymerization (ATRP) reaction can also be used to prepare multiarm polymer containing CD pendent terminal groups. Its preparation is simple and the molecular weight of the obtained CD polymer can be controlled [4]. Additionally, acrylamido-β-CD monomer with acrylamide and *N*,*N*,*N*’,*N*’-tetramethylenediamide can copolymerize by homogenous radical copolymerization [5]. In a similar process, a polymer with ferrocene pendant groups was obtained [6]. The interaction of β-CD polymeric gel with ferrocene polymeric gel and native β-CD was observed. The obtained macroscopic self-assembly system is promising for use in material science and in the medical field [7].

The second type of polymerization is the reaction of CD molecules with bifunctional agents. The most common agent is epichlorohydrin, although other epoxy compounds such as ethylene glycol, bisepoxy(propyl) ether [8] or butylene glycol bis(epoxypropyl)ether [8] have been used. These nucleophilic substitution reactions with bifunctional agents usually occur in alkaline conditions to deprotonate the hydroxyl groups of the anhydrous glucose units. The cross-linking process can create covalent chemical bonds in all directions by reaction of first and second order hydroxyl groups of cyclodextrin glucopyranose units during a polymerization or co-polymerization reaction. In this case, a macromolecular 3D network of larger size was occurred. This reaction yields the polymeric (or copolymeric) cross-linked material [9]. These new CD polymeric materials were proposed for applications in water and wastewater treatment [10,11], and were also studied for their potential applications in pharmacy and medicine [12,13,14].

CD-based nanosponges denoted as NS are spherical crosslinked nanoparticles [15]. NSs can be used in many industrial and life areas such as water purification as adsorbing agents, for agriculture as longevity enhancing agents for flowers and fruits, in fire engineering as flame retardants and smoke adsorbents, also as drug carriers and drug solubilizers in pharmaceuticals and medicine [16,17]. Nanosponges as CD polymeric materials can be obtained by polymer condensation and interfacial condensation with different crosslinkers such as: diphenyl carbonate [18], carbonyl diimidazole (CDI) [19], pyromellitic dianhydride (PMDA) [20] and hexamethylene diisocyanate (HMDI) [21]. NSs as nanoparticle-based drug delivery systems are used in pharmaceuticals to enhance the solubility, dissolution rate and stability of drugs, to convert liquid substances to solids, to mask unpleasant flavors and to prolong the release of drugs [16,22].

Synthesis of soluble CD containing polymers is a hard challenge due to the great number of reactive groups located on the CD molecule that may be involved in side reactions leading to heavily cross-linked polymers. Trotta et al. obtained innovative branched and cross-linked cyclodextrin polymers nanostructured within a three-dimensional network. This polymer system, called cyclodextrin-based nanosponges, was obtained through a direct reaction between β-CD and pyromellitic dianhydride in DMSO and Et_3_N as a weak base [17,23].

There are three types of hydroxyl groups at the cyclodextrin ring. Those at the 6th position are the most basic and often most nucleophilic, in the 2nd position are the most acidic and in the 3rd position are the most inaccessible [24]. Ring and D’Souza developed a convenient method for the functionalization of the 2nd position of cyclodextrins with sodium hydride. In anhydrous medium such as *N*,*N*-dimethyl formamide (DMF), CD easily reacts with sodium hydride (NaH) resulting in deprotonation at the hydroxyl group mainly at the C2-position of the glucopyranose unit [25]. Alternatively, by intramolecular nucleophilic substitution with the oxygen atom at the 3rd position, the CD oxyanion converts to 2,3-cyclodextrin epoxide [26].

The obtained oxyanions or epoxides react with maleic, succinic and octenylsucinic anhydrides to create CD polymers, monomers and oligomers [27,28,29,30,31]. These products can be used in medicine, food science and technology, and for removal of metal ions from diluted wastewater solution in ion flotation process because of their high solubility and anionic nature [30]. This anionic β-CD polymer crosslinked with maleic anhydride was used as a sensing material of a quartz crystal microbalance (QCM) sensor to selectively detect aromatic compounds such as benzene, toluene and *p*-xylene [32]. The same method was utilized to synthesize the β-cyclodextrin-ester network using the reaction of β-cyclodextrin with 3,3′,4,4′-benzophenone tetracarboxylic dianhydride in the DMF solution using NaH as a base to prepare CD oxyanions in the first step. The obtained β-CD-anionic polymer was used as a potential additive for poly(vinyl alcohol) (PVA)/ organo-modified layered double hydroxides (MLDH) nanocomposite film. It has been proved that the migration of tyrosine from PVA matrix for PVA/β-CD anionic polymer/MLDH composite is much lower than for PVA/MLDH matrix. In this case, the β-CD anionic polymer may improve compatibility between poly(vinyl alcohol) and synthetic anionic clays (LDH), and may stop the migration of tyrosine outside of the polymeric matrix [33].

Other CD polymers were formed by cross-linking of β-CD with nonenyl and dodecenyl derivatives of succinic anhydride, respectively and can be used in separation of metal ions by plasticized membranes [34].

In this study, water soluble, highly anionic CD polymers were prepared by cross-linking β-cyclodextrin with pyromellitic dianhydride via the oxoanion (or epoxide) intermediate prepared by the reaction with NaH. The molecular mass analysis and structural characterization of the CD-based oligomers and polymers were conducted. Solubility test and metal ion complexation process were investigated for a wide range of pH.

## 2. Materials and Methods

### 2.1. Reagents and Solvents

Crystalline β-CD, pyromellitic dianhydride (1,2,4,5-benzenetetracarboxylic dianhydride) and dimethylformamide (DMF) and cadmium (II) chloride were obtained from Sigma-Aldrich, St. Louis, MI, USA. Sodium hydride (60% suspension in paraffin oil) was obtained from MERCK (Darmstadt, Germany). Inorganic salts such as copper (II) chloride, nickel (II) chloride, calcium chloride, mercury (II) chloride, iron(III) chloride, iron(II) sulfate, silver nitrate, barium nitrate, strontium nitrate, lead(II) acetate and other solvents were kindly provided by Avantor Performance Materials Poland S.A., Gliwice, Poland. β-CD was recrystallized from water and dried for 12 h in vacuo at 110 °C. The water content was investigated using the moisture analyzer WPS-50SX, Radwag, Radom, Poland. Dimethylformamide was distilled under CaH_2_ at vacuum distilled apparatus and stored under solid CaH_2_ pellets in a dark bottle. Sodium hydride was washed with dry hexane for removing paraffin oil and used immediately. Other solvents were used without initial purification.

### 2.2. Synthesis of the CD Polymer

The typical procedure was as follows: β-CD (5.675 g, 0.005 mol) was dissolved in DMF (25 mL). Consequently, a portion of solid NaH (0.12 g (0.005 mol); 0.24 g (0.01 mol); 0.48 g (0.02 mol) or 0.96 g (0.04 mol), respectively) was slowly added into the solution. The solution was vigorous stirred over 2 h at room temperature. Subsequently, the solution solidified with many clear bubbles inside. Then solid pyromellitic dianhydride (1.09 g (0.005 mol); 2.18 g (0.01 mol); 4.36 g (0.02 mol) or 8.72 g (0.04 mol), respectively) was added. The flask was strongly shaken and the reaction mixture became a solution again. The reaction mixture was continuously stirred for the next 24 h. The product was precipitated with a large quantity of acetone, and polymer samples were extracted using acetone in Soxhlet apparatus and dried in a vacuum desiccator at room temperature to give a white crystal powder. The following weights and yields were obtained from the samples. For molar ratio 1:1:1, the weight was 6.02 g with yield 76.3%; for 1:2:2, weight was 7.26 g with yield 89.7%; for 1:4:4, weight was 8.32 g with yield 79.1%; and for 1:8:8, weight was 12.49 g with yield 81.3%. The reaction yield was calculated based on the weight of polymer samples collected after the final dry with respect to the theoretical weight which is the sum of β-CD, pyromellitic dianhydride and NaH.

^1^H NMR of the sample prepared with molar ratio 1:1:1 (600 MHz, DMSO-d_6_): 8.93 (s, 2H, benzene carboxylic); 5.74–5.63 (m, 14H, OH1 and OH3 CyD); 4.84 (d, 7H, H1 CyD); 4.46–4.38 (m, 6.22, OH6 CyD); 3.70–3.55 (m, 28H H3,H5,H6a,H6b CyD); 3.40–3.20 (m, H2 and H4 CyD overlap with water).

^1^H NMR of the sample prepared with molar ratio 1:2:2 [600 MHz, DMSO-d_6_]: 8.76 (s, 2H, benzene carboxylic); 8.45 (s, 2H, benzene carboxylate); 5.74–5.63 (m, 14H, OH1 and OH3 CyD); 4.85–4.80 (m, 7H, H1 CyD); 4.46–4.38 (m, 5.33, OH6 CyD); 3.75–3.55 (m, 28H H3,H5,H6a,H6b CyD); 3.45–3.15 (m, H2 and H4 CyD overlap with water).

^1^H NMR of the sample prepared with molar ratio 1:4:4 [600 MHz, DMSO-d_6_]: 8.86 (s, 2H, benzene carboxylic); 8.49–8.41 (m, 2H, benzene carboxylate); 5.74–5.60 (m, 13H, OH1 and OH3 CyD); 4.85–4.80 (m, 7H, H1 CyD); 4.49–4.33 (m, 4.26, OH6 CyD); 3.75–3.55 (m, 28H H3,H5,H6a,H6b CyD); 3.45–3.15 (m, H2 and H4 CyD overlap with water).

^1^H NMR of the sample prepared with molar ratio 1:8:8 [600 MHz, DMSO-d_6_]: 8.76 (s, 2H, benzene carboxylic); 8.48–8.31 (m, 2H, benzene carboxylate); 5.85–5.60 (m, 12.27H, OH1 and OH3 CyD); 4.95–4.76 (m, 7H, H1 CyD); 4.68–4.30 (m, 4.23, OH6 CyD); 3.88–3.53 (m, 28H H3, H5, H6a, H6b CyD); 3.45–3.20 (m, H2 and H4 CyD overlap with water).

### 2.3. Separation of the Polymer due to Particle Size

Whole CD polymer samples were dissolved in water and separated using the ultrafiltration process at Millipore UF Stirred Cell 76 mm with Ultrafiltration Membrane, Regenerated Cellulose PLCC 5000 Da (Merck, Darmstadt, Germany). The pressure of nitrogen gas was 2.2 bar. The separated fractions were recovered by lyophilization (Christ, Osterode am Harz, Germany) and weighed.

### 2.4. NMR Measurement

NMR spectra were recorded at 600 MHz frequency with an Avance II Bruker Ultrashield Plus Spectrometer (Bruker Polska Sp. z o.o., Poznań, Poland) and a 5 mm sample tube in DMSO-d_6_ solution without internal standard. All spectra were obtained at ambient temperature.

### 2.5. FTIR Measurement

FTIR spectra were recorded on NEXUS NICOLET FTIR spectrophotometer (Thermo Fisher Scientific, Waltham, MA, USA) in KBr disc in the range from 4000 cm^−1^ up to 400 cm^−1^. The spectra were analyzed by EZ Nexus NICOLET software (Thermo Fisher Scientific, Waltham, MA, USA).

### 2.6. DSC and TG Measurements

The thermal DSC-TG-DTG analysis was conducted with the NETZSCH STA-409 simultaneous thermal analyzer (NETZSCH Instrumenty Sp.z.o.o, Kraków, Poland), calibrated with standard indium, tin, zinc and aluminum with 99.99% purity. Samples weighing approximately 0.020 g were heated in corundum crucibles with non-hermetic lids. The heating was performed under static air conditions in the range of 20–500 °C with the 5 °C min^−1^ temperature rate. The measurements were duplicated. Accuracy of reading characteristic temperatures for DSC and TG measurements were ±0.5 °C precision in temperature reading. Recorded thermograms were analyzed with the NETZSCH-TAANALYSIS and NETZSCH SEPARATION OF PEAKS programs (NETZSCH Instrumenty Sp.z.o.o, Kraków, Poland).

### 2.7. Scaning Electron Microscopy (SEM) Measurement

VEGA3 TESCAN instrument (Tescan France, Fuveau, France) was used to obtain information of the surface characteristics of the obtained cyclodextrin polymer. Samples were used in the powdery forms. All samples were subjected to beam energy of 5 kV.

### 2.8. Molecular Mass Analysis

#### 2.8.1. HPSEC-MALLS-RI Measurement

Values of absolute molecular weight (M_w_) of water soluble β-cyclodextrin-based polymers were measured using high pressure size exclusion chromatography coupled with multiangle laser light scattering and refractometric index detectors (HPSEC-MALLS-RI). The high performance size exclusion chromatography (HPSEC) system for determining the average molecular weight consisted of a pump (Shimadzu 10AC, Tokyo, Japan), an injection valve (model 7021, Rheodyne, Palo Alto, CA, USA), a guard column TSK PWH (Tosoh Corporation, Tokyo, Japan) and connected to size exclusion column TSKgel 2500 PWXL (300 × 7.8 mm^2^, Tosoh Corporation, Tokyo, Japan). A multiangle laser light scattering detector (MALLS) operating in chromatographic mode using an He−Ne laser light source (630.0 nm) (Dawn-DSP-F, Wyatt Technology, Santa Barbara, CA, USA) and a differential refractive index detector (L-7490, Merck, Darmstadt, Germany) were connected to the columns. The columns were maintained at 30 °C. The mobile phase (0.15 M NaNO_3_ with 0.02% sodium azide) was filtered through 0.2 and 0.1 micron cellulose acetate filters (Lab-System-Service, Szczecin, Poland). The flow rates of the mobile phase and the sample injection volume were 0.4 mL/min and 300 µL, respectively. The output voltage of refractive index (RI) and light scattering (LS) at 18 angles was used for calculating the weight-average molecular weight (M_w_) using Astra 4.73.04 software (Wyatt Technology, Santa Barbara, CA, USA).

#### 2.8.2. MALDI-TOF MS Experiment

MALDI-TOF MS analyses were performed on the UltrafleXtreme (Bruker Polska Sp. z o.o., Poznań, Poland) in the linear positive ion mode at the laser frequency of 200 Hz. Calibrations were performed on protein calibration standard II from Bruker company. The matrix, 2,5-dihydroxybenzoic acid (DHB, Sigma-Aldrich, St. Louis, MI, USA), was dissolved in CH_3_CN:H_2_O:CF_3_COOH (50:50:0.1) to a final concentration of 50 mg mL^−1^. Samples (mg/mL in water) were diluted with equal volumes of matrix solution and were evaporated (0.5 μL) on a steel plate at room temperature.

### 2.9. Potentiometric and pH Experiment

The pH experiment was conducted using Multifunction Computer Metter CX-731, Elmetron, Zabrze, Poland equipped with a glass electrode. Various metal salt solutions were prepared with 0.1 mol/L concentration. The pH of these solutions was measured. A solution of 1% polymer was prepared and its pH was measured. A total of 1 mL of the polymer solution was added to 10 mL of the salt solution and the pH was measured again.

### 2.10. Solubility Experiment

Solubility measurement was conducted using the moisture analyzer WPS 50SX, Radwag, Radom, Poland (Max 50 g, d = 0.1 mg). A total of 2 g of polymer was placed in water (10 mL) and stirred using a magnetic stirrer for 30 min at room temperature. Subsequently, solution was filtered off. The filtrate (2 mL) was placed on aluminum tray and dried to dryness for 15 min at 120 °C. The weight of the residue was measured. The experiment was repeated 5 times for each sample, and the average value was calculated.

## 3. Results and Discussion

### 3.1. Synthesis of β-CDP (CDPA)

**Figure 1:**
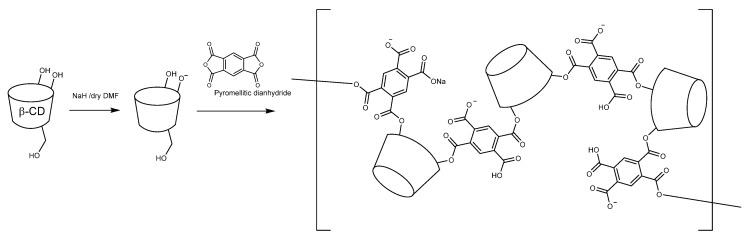
The synthesis routes of β-CD-based polymers.

In this study, pyromellitic dianhydride (PA) was used as a linker to construct highly anionic β-CD polymer. The synthesis of CDPA is shown in Figure 1. Crosslinking reaction between β-CD and PA occurs through the oxyanion intermediate prepared by NaH. In anhydrous media, such as N,N-dimethylformamide, β-CD easily reacts with sodium hydride (NaH) and the deprotonation of hydroxyl groups mainly at the C2-position of AGU molecules, leading to oxyanions [25]. Alternatively, the CD oxoanion converts to 2,3-cyclodextrin epoxide by intramolecular nucleophilic substitution with the oxygen atom at the 3-position [26].

When the molar ratio between β-CD and NaH is equal to or higher than 1:4, the gel formation is observed [31]. This gel is stiff enough to prevent stirring. After adding pyromellitic dianhydride in one portion, and strong shaking by hand, the gel was destroyed and the solution could be mixed again. The flask was also a little hot. The results regarding this phenomenon will be published soon.

The β-CD multioxyanions reacted with anhydride moieties of PA to form ester groups as a linker. The obtained β-CDPA also contains many free carboxylic groups and it is acidic (pH around 3.5).

Samples obtained in these reactions with different molar ratio, were initially separated by ultrafiltration at Milipore UF Stirres Cell equipped with Ultrafiltration Membrane with the cut-off size of 5000 Da. Table 1 represents the results of the ultrafiltration experiment.

The experiment clearly shows that when the molar ratio between CD and PA increases, the samples have higher molecular weight. When the molar ratio is 1:1:1, most of the sample contains the polymer fractions with a molecular weight of less than 5000 Da (87.00%). In this study, only around 9% of the sample contains a fraction with a molecular weight higher than 5000 Da. In case of a sample in which the molar ratio is 1:8:8, the fraction with the molecular weight is higher than 5000 Da at 40%.

### 3.2. Molecular Weight Characterization

#### 3.2.1. HPSEC-MALLS-RI Analysis

Table 2 lists the weight-average molecular weight and percent ratio of each chromatographic fraction of the polymerization products under different molar ratio.

**Figure 2:**
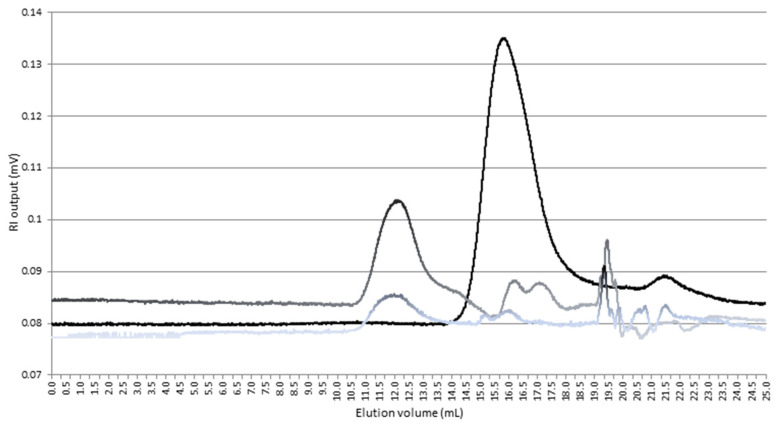
High pressure size exclusion chromatography (HPSEC) chromatograms of β-CD polymerization products prepared at molar ratio of β-CD:NaH:PA (1:1:1).

All native samples and samples obtained during the ultrafiltration process were investigated. Each reaction product obtained with a different molar ratio consisted of several peaks with distinctively different *M*w values under a high-performance size-exclusion chromatography. The reaction occurred in heterogeneous or discontinuous patterns. However, all samples prepared with different molar ratios have a similar pattern of the chromatogram, and the chromatogram of each sample consists of two major groups of peaks. One with M_w_ greater than 10^4^ Da was detected with elution volume average 11.30 to 12.90 mL, and the second with M_w_ from 3000 up to 9000 Da with an elution volume average between 14.00 to 16.50 mL (Figure 2). In all cases, regardless of the molar ratio of the reactants, fractions cut off below 5000 Da show a lot of similarities. Their characteristic features are high polydispersity (>5 M_W_/M_N_) which clearly indicates mixture of many CD derivatives systems with small average molecular weights, such as CD derivatives, dimers and small oligomers composed of at most three or four CD molecules

CD polymers obtained during ultrafiltration process with higher average molecular weights (fractions cut off up 5 kDa) are characterized by low polydispersity, between 1.03 and 1.89 M_W_/M_N_.

In all cases, these obtained systems have average molecular weights above 10,000 Da and when more hydroxyl groups are activated in the CD molecule (molar ratio of 1:8:8 reagents), even polymer molecules with average masses above 100,000 Da have also been observed.

It is not possible to accurately determine the average molecular mass for small molecules due to the low resolution of the chromatographic technique used in HPSEC-MALLS-RI system.

Based on the *M*w distribution of the β-CD polymers obtained for reagents with different molar ratio, it can be concluded that NaH produces mono- and multioxyanions on a β-CD molecule, depending on the amount of NaH used in the reaction. The multioxyanionic β-CD derivatives could react with more molecules of pyromellitic anhydride, and form polymers with benzene moiety ester bridges. Therefore, molar ratio of the reagents plays an important role in determining the structure and molecular weight of the CD polymer.

#### 3.2.2. MALDI-TOF Experiment

The analysis of MALDI-TOF spectra for samples with different molar ratios was performed to better understand the polymer structure and to determine how the molar ratio of reactants affects the formation of CD-based polymers.

To observe all the possible polymer fragments, only native samples were analyzed. Regardless of sample preparation, the fragment with molar mass 1157, assigned to [β-CD-Na^+^], was observed. There were no other signals at a lower molecular weight than β-CD [35].

Most of the peaks which are observed in signal groups corresponding to small polycyclodextrin fragments appear at intervals between 236 to 239 which can be addressed to pyromellitic moieties of linker or monomer [PA-Na^+^]. Conversely, different values of intervals allow the assumption that some PA moieties could be used as a linker between cyclodextrin molecules, but others are only substituents directly connected to cyclodextrin. Consequently, the obtained CD anionic polymers have a branched β-CD structure rather than cross-linked CD polymeric structure.

In most cases, many permutations and combinations of molecular configuration between CD molecules and PA linkers and PA moieties have been observed. However, regularity can be observed for systems built from 2 to 5 CD molecules. When the number of CD molecules increase, the number of pyromellitic linkers and moieties also proportionally increase. Table 3 presents the calculations of molecular masses for that fragments pressure.

It is obvious that when molar ratio of the reagents increased, also substitution of β-CD by pyromellitic groups increased. It take notice that the molar ratio of the reagents slightly influenced on the branched structure of CD polymers. However, when molar ratio increased (1:4 or 1:8), the oligomeric fragments with 3 to 5 CD molecules are also observed. When the molar ratio is 1:1 or 1:2, only small fragments with 1 or 2 CD molecules are indicated.

**Figure 3:**
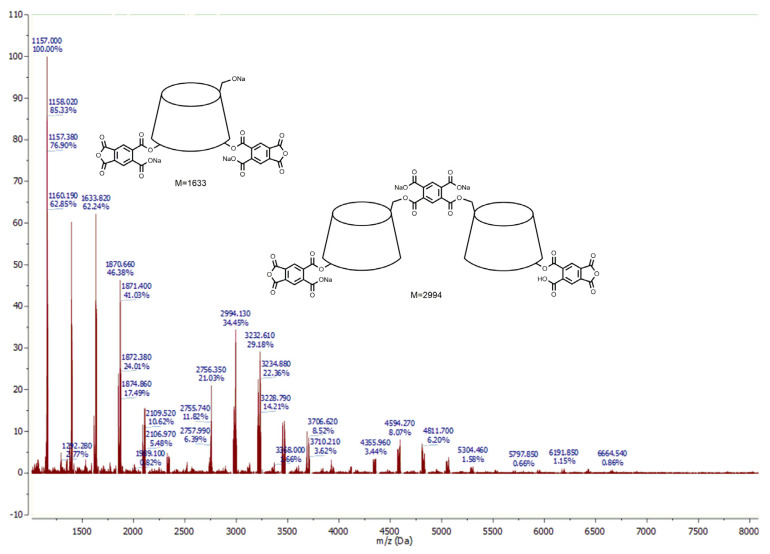
MALDI-TOF spectra of the products of polymerization reaction of CD oxyanions with pyromellitic dianhydride with molar ratio of the reagents 1:4:4.

**Figure 4:**
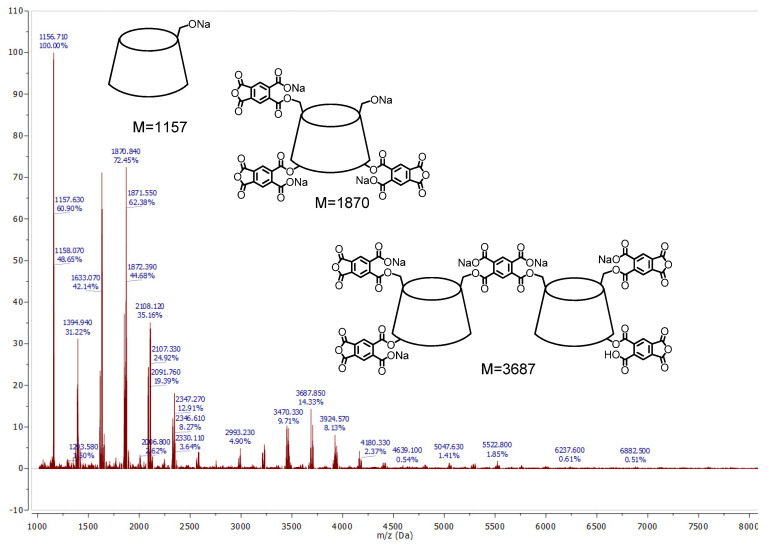
MALDI-TOF spectra of the products of polymerization reaction of CD oxyanions with pyromellitic dianhydride with molar ratio of the reagents 1:8:8.

Figure 3 and Figure 4 show the molecular structure analysis of sample prepared with molar ratio 1:4:4 and 1:8:8, respectively. Other samples have a similar pattern.

When the molar ratio between reagents increased, the number of pyromellitic moieties directly substituted to CD molecules also increased, but the number of pyromellitic linkers remained unchanged. This phenomena can best be seen for the group signals corresponding to dimeric CDs with one pyromellitic linker. When the molar ratio is 1:4:4, the main peak in the group signal corresponds to 2CD + L + 2M + 3Na molecule with molecular mass equal to 2994. In the case of molar ratio 1:8:8, the main peak corresponds to 2CD + L + 5M + 5Na molecule with molecular mass equal to 3687.

Lack of full correspondence between calculated and observed molecular weights for branched β-CD fragments could be explained by the possibility of formation of inclusion complexes between CD molecules and small molecules such as water or DMF, which can play the role as guest or passenger molecules. This phenomena was observed in the case of polymerizable cyclodextrin derivatives analyzed by MALDI-TOF technics by Bowen and coworkers [36].

Anionic polymeric fragments with higher molecular weight, which were detected by GPC chromatography measurements, in the case of MALDI-TOF technics are not observed, similar to the earlier reported investigations on CD oligomers crosslinked by epichlorhydrin and choline chloride or chloroacetic acid [37].

### 3.3. FTIR Characterization

**Figure 5:**
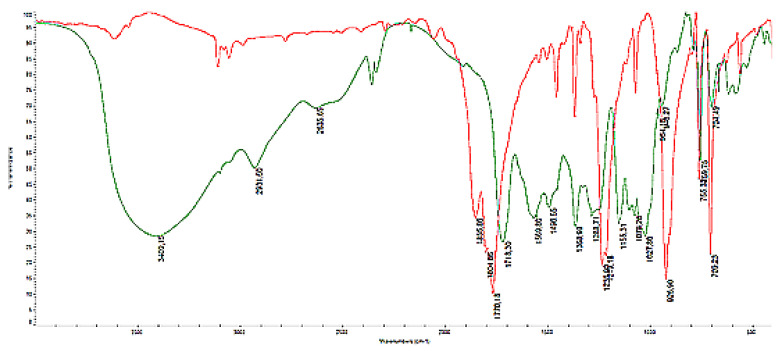
FTIR spectra of pyromellitic dianhydride and branched β-CD polymer obtained with 1:8:8 ratio.

The polymerization reaction between β-CD and PA in the presence of NaH is confirmed by FTIR spectroscopy (Figure 5). The ring opening reaction of anhydride moiety of PA is observed. The absorption band of carbonyl group (C=O stretching) at 1770 cm^−1^ is shifted to 1718 cm^−1^ in the β-CD polymer. The O–H stretching band (centered at 3409 cm^−1^) is broad because it can be assigned to primary and secondary hydroxyl groups of CD and also to OH stretching which are formed after opening the cyclic anhydride moiety included in PA. When the oxyanions or epoxides are formed on secondary faced cyclodextrin rim, the branched polymer could be created rather than the cross-linked polymer in which also primary hydroxyl groups can be consumed. A similar correlation was observed by Trotta and coworkers [23]. In association with the main OH stretching bands are also the bands at 2500–2700 cm^−1^ (centered at 2635 cm^−1^) which can be assigned to overtones of bands located at 1200–1500 cm^−1^ which are assigned to OH bending and C-O stretching of carboxyl groups.

### 3.4. NMR Investigation

NMR measurement of CD-based polymers obtained with various molar ratios of reactants was carried out. ^1^H NMR spectra are consistent with the proposed reaction for carboxylic ester formation. The degree of substitution of CD molecules based on the number of primary and secondary hydroxyl groups involved in the polymerization reaction was analyzed. For CD-based polymer prepared with 1:2:2 molar ratio, only 1 or 2 primary hydroxyl groups are involved in the formation of ester bonds. The ratio between anomeric proton H-1 and OH-6 of AGU is equal to 7:5.33. For oligomers and polymers prepared with higher molecular ratios such as 1:4:4 and 1:8:8, secondary hydroxyl groups are involved in this reaction. For CD-based polymer prepared with 1:4:4 molar ratio, 2 or 3 primary hydroxyl groups were reacted and the ratio between anomeric proton H-1 and OH-6 of AGU is equal to 7:4.26:12.89. In case of CD-based polymer prepared with 1:8:8 molar ratio, secondary hydroxyl groups of AGU are involved in the reaction. For this reaction ratio of anomeric proton H-1, primary OH-6 and secondary hydroxyl groups OH-2 or OH-3 equal to 7:4.23:12.27 is observed. It can be explained that the CD was not completely dried and contained traces of water molecules. In this case, the sodium hydride can produce traces of hydroxide that activates primary hydroxyl groups. At higher molar ratios of reagents (1:4:4 and 1:8:8), the expected activation of secondary hydroxyl groups occurs. In the aromatic region, the signal of benzene proton at 7.95 ppm is observed regardless of the molar ratio of reactants. This signal is assigned to unreacted 1,2,3,4-tetrabenzenecarboxylic acid or its sodium salt which can be formed from pyromellitic dianhydride in this strong basic reaction conditions. Other signals at 8.76 and 8.45 ppm are assigned to benzene protons of carboxylic and carboxylate moieties at CD-based oligomers and polymers. The integration ratio between anomeric proton H-1 of AGU and benzene protons of linker moieties are in good agreement with the observation that the molar ratio of the reactants is higher in the case when more benzene carboxylate linkers were created.

### 3.5. TG/DSC Investigations

**Figure 6:**
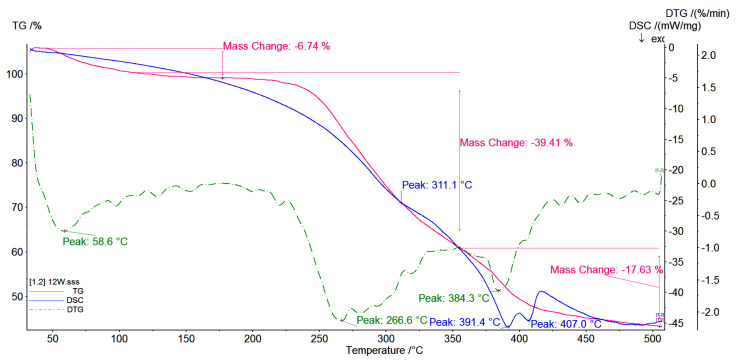
TG/DTG/DSC curves of the products of the polymerization reaction of CD oxyanions with pyromellitic dianhydride with molar ratio of the reagents 1:2:2.

**Figure 7:**
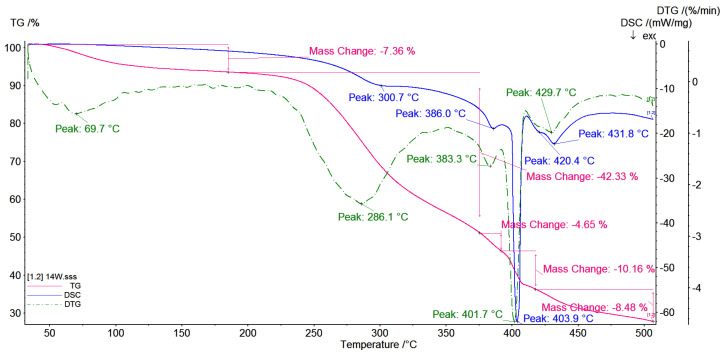
TG/DTG/DSC curves of the products of the polymerization reaction of CD oxyanions with pyromellitic dianhydride with molar ratio of the reagents 1:4:4.

**Figure 8:**
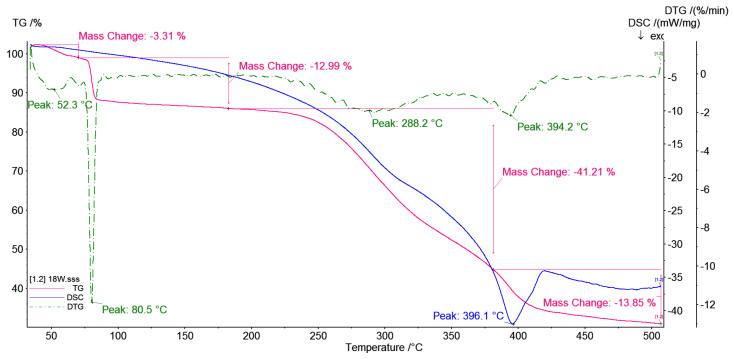
TG/DTG/DSC curves of the products of the polymerization reaction of CD oxyanions with pyromellitic dianhydride with molar ratio of the reagents 1:8:8.

Thermal analysis (DSC and TG) of native β-CD, pyrromelitic dianhydride (PA) and CD-based polymers obtained with various molar ratio of reactants was carried out. In all observed polymer thermograms, the removal of hydrated water is observed. The course of TG curves for polymers obtained with 1:2:2 (Figure 6) and 1:4:4 (Figure 7) molar ratios are very similar. Both of them contain a small amount of water absorbed on the surface of branched cyclodextrin oligomers and small mass loss, approximately 7%, is observed. The mass loss corresponds with the slight thermal effects at approx. 60 °C. In addition, we can see that speed of water lost for CD-based polymer prepared with 1:2:2 molar ratio is higher. The slope of the TG line, i.e., tgα is equal to 1.7 (the slope is defined as a mass loss percent (%) for 1 °C) than for polymers prepared with higher molar ratios of 1:4:4 and 1:8:8, tgα = 1.1 and tgα = 1.4, respectively. The increase of molecular mass of the system causes that the water content decreases, 7% for CD-based polymer prepared with 1:4:4 molar ratio and 3% for CD-based polymer prepared with 1:8:8 molar ratio (Figure 8). In all the cases, the speed of mass lost is similar. The analysis of TG and DSC curves indicates that for the CD oligomer with average smallest molecular mass (for samples with molar ratio 1:2:2), the water molecules associated with surface are weaker than for other CD oligomers or polymers. In this case, sorption from the surface is more effective than for polymers prepared with higher molar ratio.

For the CD oligomer prepared with molar ratio 1:4:4, the water lost (7%) is also observed, but in this case the temperature of water loss is 10 degrees higher than for CD oligomers prepared with molar ratio 1:2:2. It means that water molecules are embedded deeper within the branched polymer arms. In the case of CD polymer with higher molecular mass (molar ratio 1:8:8), the water loss is a two-step process. The first stage is observed at the temperature 60 °C, such as for the other obtained systems. It is also water molecules absorbed on the surface of the polymer and the speed of the water loss is similar like in oligomers with smaller molecular mass and is equal to tgα = 1.4. The second stage is observed at 80 °C. Water loss is observed with tgα = 5.57 and equal to 13% of total mass of the sample, and this mass loss corresponds to loss of water more deeply embedded in the branched polymer structure than in the oligomer or polymer with less molecular mass. The water loss observed on TG curves is mainly water which is absorbed inside the branched polymer arms not only inside the CD cavity.

In all events of all analyzed systems, the plateau up to approximately 250 °C is observed. There are no visible thermal effects on the DSC curves that correspond to phase transitions.

When temperature is raised above 250 °C, the decomposition of all analyzed systems is observed.

The onset temperature for CD-based polymer prepared with 1:2:2 molar ratio is 244.3 °C, for polymer prepared with 1:4:4 it is 246.6 °C and for polymer prepared with 1:8:8 it is 250.9 °C. The analysis of TG curves shows that the degree of branching of the polymer increases. In all cases in the range of temperature 250–370 °C only one weight loss (~40%) with very similar rate of decomposition is observed. The speed of weight loss for CD-based polymer prepared with 1:2:2 molar ratio is equal to tgα = 1.9, for polymer prepared with 1:4:4 it is equal to tgα = 2.0 and for polymer prepared with 1:8:8 it is equal to tgα = 1.95.

In all cases, the exothermic effects have a maximum at temperature around 310 °C. When the molecular mass of the polymers increases, the weight loss is higher. For polymers with a more branched structure, faster and higher weight loss is observed. It was noted that systems containing more branched structures are less stable.

It is noticed that anionic highly branched polymers form amorphous rather than crystalline systems (see the amylopectin structure) [38]; thus, the thermal stability of such systems decreases with the increase of molecular mass.

The CD-based polymer thermograms are not the superposition of the cyclodextrin and pyromellitic dianhydride spectra. This fact could indicate the formation of a polymer in which the cyclodextrin rings are joined together by a linker. The TG/DTG curves also confirm the above observations.

### 3.6. SEM Investigation

The morphological studies were performed by SEM to check the surface porosity of the obtained polymers. Porous materials have been used in various areas such as water and gas purification or catalysis.

**Figure 9:**
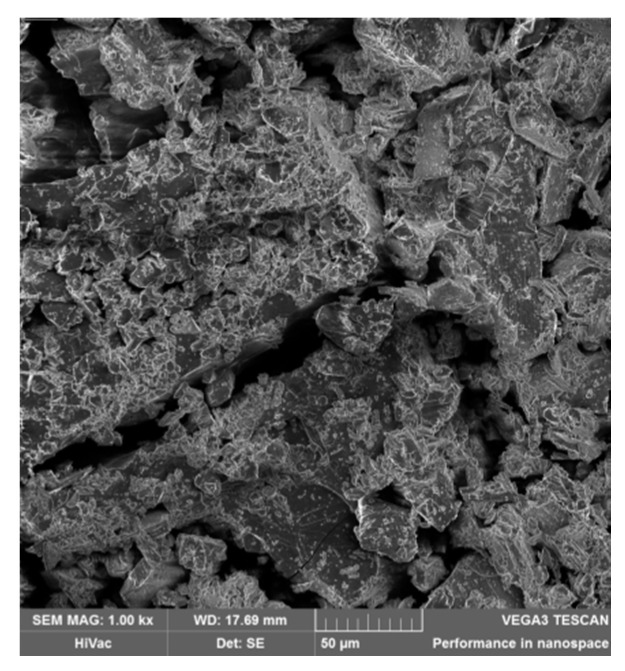
Sample containing the β-CD monomer structure.

Figure 9 shows the crystalline structure of the cyclodextrin monomer. A non-smooth surface with numerous protuberances and cracks, which can be assigned to the crystal structure of cyclodextrin, is observed.

**Figure 10:**
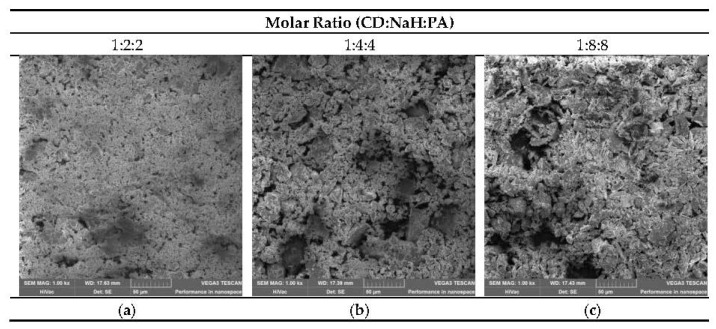
(**a**–**c**): Samples containing the polymer fractions with molecular weight less than 5000 Da.

Figure 10a-c show SEM micrographs of the polymer fractions with molecular weight less than 5000 Da. The surface structure is totally different than the crystal structure of cyclodextrin monomer. In all cases, it can be seen that the polymer particles are closely packed into the polymer network. Many mesopores and macropores in the structure are observed. The packing pattern tends to vary according to the increase in the molar ratio between CD and PA. Fractions with molecular weight less then 5kDa are very similar but the oligomer with molar ratio 1:8:8 between CD, NaH and PA (Figure 10c) has a surface with not so closely packed material and with bigger macropores than the surface of other oligomers (Figure 10a,b).

**Figure 11:**
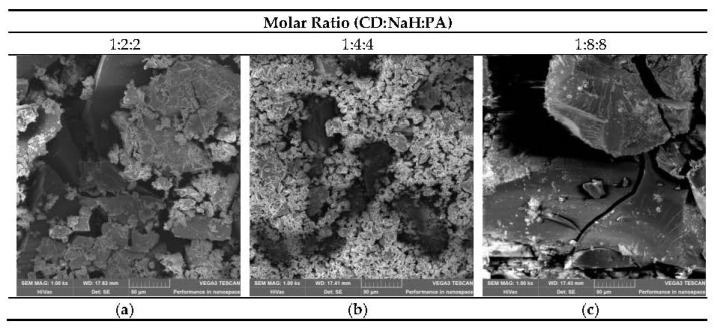
(**a**–**c**): Samples contain the polymer fractions with molecular weight higher than 5000 Da.

Figure 11a–c show SEM micrographs of the polymer fractions with molecular weight higher than 5000 Da. Structures with a tightly packed surface can be observed in the structure of polymer fractions with molecular weights greater than 5000 Da. For the polymers obtained with molar ratio 1:8:8, the very smooth surface with particles that are closely packed is observed. Similar parts of smooth surfaces are observed in other samples, but structures with tightly packed surfaces are also noticed.

It seems that samples with molecular weights less than 5000 Da may have higher sorption properties than polymers with higher molecular weights where more uniform, pore-free surfaces were observed.

### 3.7. Solubility in Water

The synthesized β-CD polymers obtained with different molar ratio have high solubility in water. It is also observed that solubility strongly depends on molecular weight of the polymer samples. The samples with molecular weight higher than 5000 Da were more soluble than samples with molecular weight lower than 5000 Da. Results of the solubility experiment are shown in Table 4.

The high solubility of the fractions with high molecular weight depends on the presence of a large amount of free carboxyl groups. It means that β-CD-based polymer is more branched than cross-linked. Another confirmation of this assumption is the dependence of the solubility of the polymer on pH. When pH is below 1 (strongly acidic), the precipitation of the β-CD polymer is observed. In this case, free carboxylic groups are protonated. We also can observe the reverse process. When the pH is neutral the precipitated polymer is dissolved again.

### 3.8. Potentiometric and pH Experiment

Metal salt solutions with concentration 0.1 mol/L were prepared and the pH was measured. A total of 1 mL of the polymer solution was added to 10 mL of the salt solution and the pH was measured again. Results are shown in Table 5. The pH of 1% β-CD-based polymer (1:8:8 molar ratio, whole sample) was equal to 3.49.

Water solution of obtained β-CD polymer (1:8:8 molar ratio, whole sample) can precipitate in the presence of selected metal ions: Fe^2+^, Fe^3+^, Pb^2+^ and Ag^+^. In this case, the metal–organic complex is formed. In case of other ions, such as: Cd^2+^, Cu^2+^, Ca^+2^, Ba^2+^, Hg^2+^, Ni^2+^ and Sr^2+^, the precipitation is not observed. The similar complexation phenomena was observed for branched β-CD polymer (1:8:8 molar ratio, >5 kDa fraction). In this case, slight precipitation for Sr^+2^ ion complexation was also observed. This evidence shows some selectivity towards certain metal cations and indirect evidence of polyelectrolyte behavior of β-CD-based polymer. No effect of anions on cation selectivity has been observed.

## 4. Conclusions

Water soluble, highly anionic CD polymers were prepared by cross-linking β-cyclodextrin with pyromellitic dianhydride via the oxyanion (or epoxide) intermediate prepared by the reaction with NaH. Different molar ratios of the reagents were investigated in terms of molecular size, chemical structure and water solubility of the polymers. The separation of the polymer due to particle size by ultrafiltration process and HPSEC-MALLS-RI and MALDI-TOF MS measurements for molecular mass analysis were employed. The IR, HNMR, DSC and TG measurements for the structural characterization of the polymers were conducted. The H NMR investigation showed very interesting phenomena. Despite the use of sodium hydride for activation of secondary OH group at the 2-nd position in AGU, the crosslinking reaction between CD monomer and pyromellitic dianhydride proceeds also via a primary OH group at the 6-th position. It can be explained that the CD was not completely dried and contained traces of water molecules. In this case, the sodium hydride can produce traces of hydroxide that activates primary hydroxyl groups. At higher molar ratios of reagents (1:4:4 and 1:8:8), the expected activation of secondary hydroxyl groups occurs. The structure of obtained polymers remains unresolved and needs more investigations. On the one hand, FTIR spectroscopy suggests a predominantly branched structure, however the partial consumption of the primary OH groups allows to assume the cross-linked structure. Finally, the solubility test and metal ion complexation process in a wide range of pH were also investigated. The anionic CD-based polymer has a polyelectrolyte structure and can be employed in selective indication and separation of many heavy metal anions from waste water. The highly polyanionic structure and high molecular mass of obtained CD-based polymers with highly water solubility, are promising for use in pharmaceutical industry for drug solubilization or encapsulation. They are also promising for the protection of degradable substances or in the synthesis of new drug delivery systems.

## Figures and Tables

**Table 1:** Ultrafiltration data for samples prepared with different molar ratios of β-CD, NaH and pyromellitic dianhydride.

Molar Ratio (CD:NaH:PA)	Cut-Off	Weight	% of Whole Sample
1:1:1	Whole sample	3.00 g	100.00%
	<5000 Da	2.63 g	87.00%
	>5000 Da	0.26 g	8.67%
1:2:2	Whole sample	4.00 g	100.00%
	<5000 Da	3.04 g	76.00%
	>5000 Da	0.59 g	14.75%
1:4:4	Whole sample	4.00 g	100.00%
	<5000 Da	2.07 g	51.75%
	>5000 Da	0.99 g	24.75%
1:8:8	Whole sample	4.00 g	100.00%
	<5000 Da	1.84 g	46.00%
	>5000 Da	1.60 g	40.00%

**Table 2:** Weight-average molecular weight and polydispersity ratio of the β-CD polymerization products prepared at different molar ratio of β-CD:NaH:PA.

Molar Ratio (β-CD:NaH:PA)		Weight-Average Molecular Weight (M_W_)	Polydispersity (M_w_/M_n_)
1:1:1	(Whole sample)	6.83 × 10^4^	1.08
		8.62 × 10^3^	1.12
	(<5000 Da)	3.87 × 10^3^	7.93
	(>5000 Da)	1.12 × 10^4^	1.07
		5.13 × 10^3^	3.39
1:2:2	(Whole sample)	2.84 × 10^4^	1.32
		2.13 × 10^4^	2.20
	(<5000 Da)	1.011 × 10^4^	12.3
	(>5000 Da)	8.06 × 10^4^	1.19
		3.38 × 10^3^	1.89
1:4:4	(Whole sample)	8.84 × 10^4^	1.34
		2.77 × 10^3^	1.27
	(<5000 Da)	9.89 × 10^3^	8.34
	(>5000 Da)	8.91 × 10^4^	1.17
		2.30 × 10^4^	1.03
1:8:8	(Whole sample)	6.65 × 10^5^	1.13
		8.22 × 10^4^	1.70
		1.23 × 10^4^	1.39
	(<5000 Da)	5.49 × 10^3^	5.07
	(>5000 Da)	6.22 × 10^5^	1.18
		5.96 × 10^3^	1.03

**Table 3:** Approximate molecular weights of cyclodextrin polymeric fragments detected at the MALDI-TOF experiment. [CD]—cyclodextrin molecule; [L]—pyromellitic linker; [M]—pyromellitic moiety; [Na]—sodium cation.

Structure of Anionic Polymer Fragment	Calculated Molecular Masses	Observed Molecular Masses
CD+Na	1157	1157
CD + M + 2Na	1397	1395
CD + 2M + 3Na	1637	1632
CD + 3M + 4Na	1876	1870
2CD + L + M + 2Na	2749	2756
2CD + L + 2M + 3Na	2989	2994
2CD + L + 3M + 4Na	3229	3232
2CD + L + 5M + 5Na	3687	3687
3CD + 2L + 2M + 2Na	4348	4355
3CD + 2L + 3M + 4Na	4589	4594
3CD + 2L + 4M + 4Na	4829	4832
4CD + 3L + 4M + 4Na	6195	6191
5CD + 4L + 5M +4 Na	7780	7783

**Table 4:** Sample solubility prepared with different molar ratios of β-CD, NaH and pyromellitic dianhydride.

Molar Ratio (CD:NaH:PA)	Whole Sample [mg/mL]	<5000 Da [mg/mL]	>5000 Da [mg/mL]
1:1:1	28.7	20.4	91.1
1:2:2	83.1	79.4	157.1
1:4:4	85.4	62.9	171.3
1:8:8	75.6	42.8	172.1

**Table 5:** pH measurement of 0.1 mol/L solution of various metal salts before and after adding the β-CD branched polymer.

Metal Ion	pH of Salt Solution	pH of Salt + Polymer Solution	Comments
Fe^2+^	3.85	3.40	Precipitation
Cd^2+^	6.84	3.24	
Cu^2+^	4.28	3.04	
Ca^2+^	7.81	3.21	
Ba^2+^	6.58	3.24	
Hg^2+^	4.06	3.48	
Ni^2+^	7.30	3.21	
Ag^+^	10.51	9.25	Precipitation
Pb^2+^	7.01	5.64	Precipitation
Fe^3+^	2.29	2.35	Precipitation
Sr^2+^	6.84	3.26	
